# Crystal structure of 4-methyl-*N*-{(*E*)-meth­yl[(3a*R*,8a*S*)-2-oxo-3,3a,8,8a-tetra­hydro-2*H*-indeno­[1,2-*d*][1,3]oxazol-3-yl]-λ^4^-sulfanyl­idene}benzene­sulfonamide

**DOI:** 10.1107/S2056989015024779

**Published:** 2015-12-31

**Authors:** Patrícia A. Pereira, Bruce C. Noll, Allen G. Oliver, Gustavo P. Silveira

**Affiliations:** a Universidade Federal do Rio Grande do Sul, Instituto de Química Depto. Química Orgânica, Av. Bento Gonçalves, 9500 Agronomia, Porto Alegre RS 91501-970, Brazil; b Bruker AXS Inc., 3456 E. Cheryl Parkway, Madison, WI 53711, USA; c Department of Chemistry & Biochemistry, University of Notre Dame, Notre Dame IN 46556, USA

**Keywords:** oxazolidinone, vinyl sulfonamide, crystal structure

## Abstract

The formulation that the title compound, C_18_H_18_N_2_O_4_S_2_, adopts is a zwitterionic core with the charge separated to the sulfilimine S and N atoms and is supported by the two different S—N bond distances about the sulfinimine N atom [1.594 (2) and 1.631 (2) Å, respectively] that are typical for such bonds. The notably unusual bond is S—N(oxazolidinone) [1.692 (2) Å] that is longer than a typical S—N bond [1.603 (18) Å, *Mogul* analysis; Macrae *et al.* (2008 ▸). *J. Appl. Cryst.* **41**, 466–470]. The bond-angle sum about sulfilimine sulfur (308.35°) reflects the trigonal–pyramidal geometry of this atom. Two of the angles are less than 100°. Despite the pyramidalization of this sulfur, there are no significant inter­molecular inter­actions, beyond usual van der Waals contacts, in the crystal packing.

## Related literature   

Oxazolidinone sulfilimines are synthesized as precursors of vinyl sulfilimines which are used in the γ-lactamization reaction to generate chiral pyrrolidinones with medicinal chemistry inter­est. For the synthesis, see: Celentano & Colonna (1998 ▸); Silveira & Marino (2013 ▸). For sulfonyl oxazolidinone structures, see: Barbey *et al.* (2012 ▸); Berredjem *et al.* (2010 ▸); Bonnaud *et al.* (1987 ▸); Dewynter *et al.* (1997 ▸). For related vinyl sulfonamide chemistry, see: Silveira *et al.* (2013 ▸). For related oxazolidinone sulfinime structures, see: Silveira *et al.* (2012 ▸, 2014 ▸). For the Hooft parameter, see: Hooft *et al.* (2008 ▸).
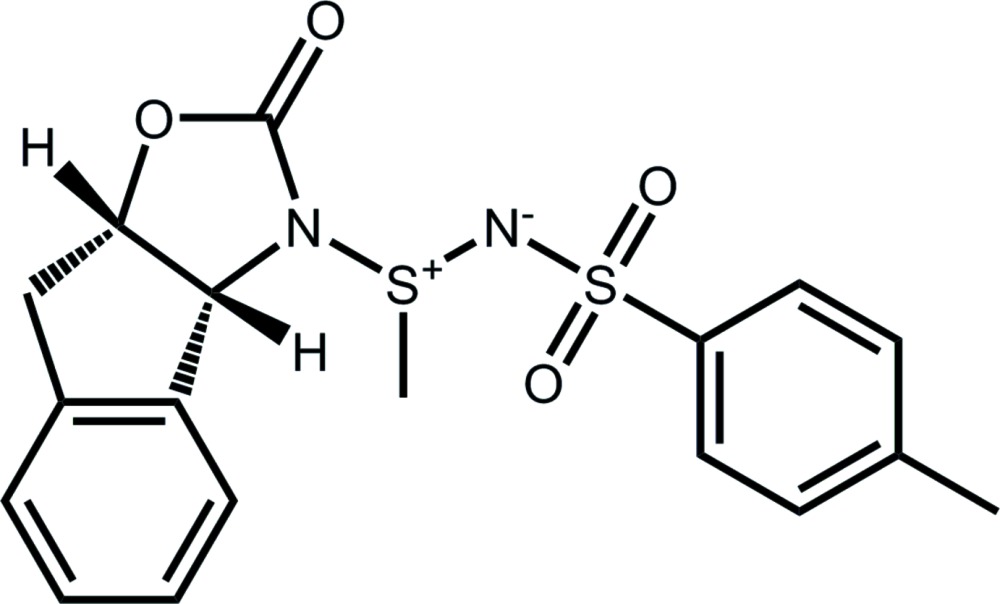


## Experimental   

### Crystal data  

C_18_H_18_N_2_O_4_S_2_
*M* *_r_* = 390.46
Orthorhombic, 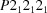

*a* = 6.8841 (1) Å
*b* = 12.2326 (2) Å
*c* = 20.1911 (4) Å
*V* = 1700.30 (5) Å^3^
*Z* = 4
Cu *K*α radiation
μ = 3.09 mm^−1^
*T* = 100 K
0.42 × 0.40 × 0.34 mm

### Data collection  

Bruker SMART APEX CCD diffractometer
Absorption correction: multi-scan (*SADABS*; Krause *et al.*, 2015 ▸) *T*_min_ = 0.701, *T*_max_ = 0.929
11963 measured reflections
3004 independent reflections
2984 reflections with *I* > 2σ(*I*)
*R*_int_ = 0.032

### Refinement  

*R*[*F* ^2^ > 2σ(*F* ^2^)] = 0.028
*wR*(*F* ^2^) = 0.070
*S* = 1.11
3004 reflections
237 parameters
H-atom parameters constrained
Δρ_max_ = 0.29 e Å^−3^
Δρ_min_ = −0.36 e Å^−3^
Absolute structure: Flack *x* determined using 1183 quotients [(*I* ^+^)−(*I* ^−^)]/[(*I* ^+^)+(*I* ^−^)] (Parsons *et al.*, 2013 ▸)
Absolute structure parameter: 0.052 (4)

### 

Data collection: *APEX2* (Bruker, 2007 ▸); cell refinement: *APEX2* and *SAINT* (Bruker, 2007 ▸); data reduction: *SAINT* and *XPREP* (Bruker, 2007 ▸); program(s) used to solve structure: *SHELXS97* (Sheldrick, 2008 ▸); program(s) used to refine structure: *SHELXL2014* (Sheldrick, 2015 ▸); molecular graphics: *XP* in *SHELXTL* (Sheldrick, 2008 ▸); software used to prepare material for publication: *XCIF* (Sheldrick, 2008 ▸) and *publCIF* (Westrip, 2010 ▸).

## Supplementary Material

Crystal structure: contains datablock(s) I, global. DOI: 10.1107/S2056989015024779/nk2234sup1.cif

Structure factors: contains datablock(s) I. DOI: 10.1107/S2056989015024779/nk2234Isup2.hkl

Supporting information file. DOI: 10.1107/S2056989015024779/nk2234Isup3.cml

**Fig. 1.**
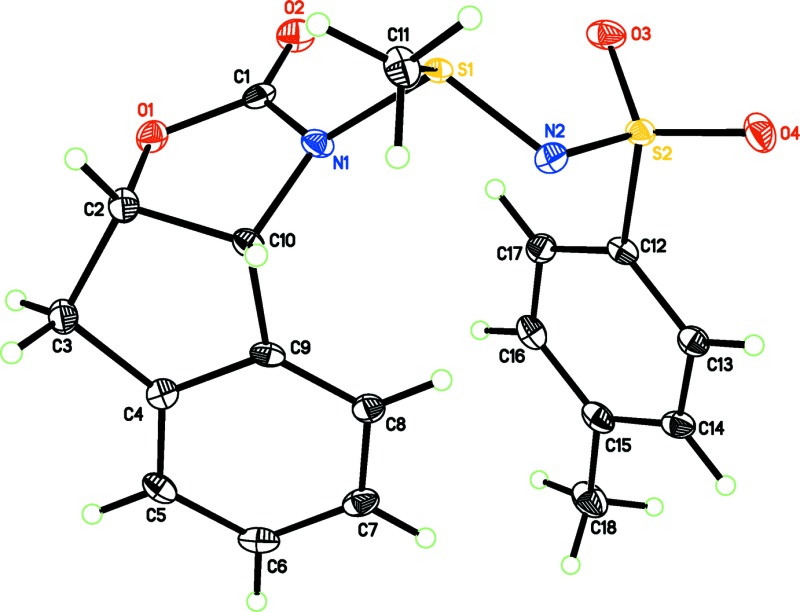
Labeling diagram of the title compound. Atomic displacement ellipsoids depicted at the 50% probability level. Hydrogen atoms depicted as spheres of an arbitrary radius.

. DOI: 10.1107/S2056989015024779/nk2234fig1.tif
Labeling diagram of the title compound. Atomic displacement ellipsoids depicted at the 50% probability level. Hydrogen atoms depicted as spheres of an arbitrary radius.

CCDC reference: 1444186

Additional supporting information:  crystallographic information; 3D view; checkCIF report

## Figures and Tables

**Table:** 

S1—N2	1.594 (2)
S1—N1	1.692 (2)
S1—C11	1.782 (3)
S2—O4	1.4430 (19)
S2—O3	1.451 (2)
S2—N2	1.631 (2)
S2—C12	1.768 (2)

**Table:** 

N2—S1—N1	110.58 (11)
N2—S1—C11	99.43 (12)
N1—S1—C11	98.34 (11)
O4—S2—O3	117.24 (12)
O4—S2—N2	106.79 (12)
O3—S2—N2	112.27 (11)
O4—S2—C12	107.93 (11)
O3—S2—C12	108.03 (12)
N2—S2—C12	103.67 (12)

## supplementary crystallographic information

### S1. Commentary

Oxazolidinone sulfilimines are synthesized as precursors of vinyl sulfilimines which are used in the γ-la­cta­mization reaction (Silveira & Marino, 2013) to generate chiral pyrrolidinones with medicinal chemistry inter­est. This compound represents one of only five other oxazolidinone compounds that incorporate an N—S—N backbone (Barbey *et al.*, 2012; Berredjem *et al.*, 2010; Bonnaud *et al.*, 1987; Dewynter *et al.*, 1997). It is the only reported structure that does not have a sulfonyl bridging the oxazolidinone ring to the second nitro­gen atom. Related sulfur-containing oxazolidinones have been reported previously by us (Silveira *et al.*, 2012; 2014). We have also reported a related sulfur-containing indole as a precursor of physostigmine alkaloid·(Silveira *et al.*, 2013).

### S2. Synthesis and crystallization

Following literature preparative methods (Celentano *et al.*, 1998; Silveira & Marino, 2013): chloramine-T (951 mg, 4.18 mmol) and hexa­decyl­tri­butyl­phospho­nium bromide (100 mg, 0.20 mmol) were added to a solution of oxazolidinone sulfide (840 mg, 3.80 mmol) in toluene (25 ml) at 295 K. After overnight stirring, ethyl acetate (25 ml) was added and the mixture washed (2 *x* 30 ml water; 1 *x* 30 ml brine), dried over anhydrous sodium sulfate, filtered, and the mixture of solvents rota-evaporated at reduced pressure. To the resulting dry, crude product, silica gel (6 g) and methyl­ene chloride (5 ml) were added and this slurry was rota-evaporated to yield the crude product on silica. This mixture was separated and purified through flash chromatography with elution (70% ethyl acetate / 30% hexanes). After slow evaporation of the ethyl acetate / hexanes elutant, the more polar sulfilimine was obtained as white crystals (688 mg). Two sulfilimine diasterioisomers were obtained in a ratio of 2.5:1 (total of 65% yield).

### S3. Refinement details

The structure was modeled routinely with all non-hydrogen atoms included with an anistropic model. Hydrogen atoms were included in calculated positions riding on the atom to which they are bonded (C—H = 0.99 Å for methyl, 0.95 Å for methyne and aromatic. *U*_iso_(H) was set = 1.5*U*_eq_(C) for methyl and 1.2*U*_eq_(C) for all others.

The absolute configuration from synthesis was confirmed by comparison of intensities of Friedel pairs of reflections yielding a Flack *x* parameter = 0.052 (4) (Parsons *et al.*, 2013) and a Hooft *y* parameter = 0.050 (6) (Hooft *et al.*, 2008).

### Crystal data

**Table:**

C_18_H_18_N_2_O_4_S_2_	*D*_x_ = 1.525 Mg m^−^^3^
*M**_r_* = 390.46	Cu *K*α radiation, λ = 1.54178 Å
Orthorhombic, *P*2_1_2_1_2_1_	Cell parameters from 9988 reflections
*a* = 6.8841 (1) Å	θ = 4.2–69.2°
*b* = 12.2326 (2) Å	µ = 3.09 mm^−^^1^
*c* = 20.1911 (4) Å	*T* = 100 K
*V* = 1700.30 (5) Å^3^	Parallelepiped, clear colorless
*Z* = 4	0.42 × 0.40 × 0.34 mm
*F*(000) = 816	

### Data collection

**Table:**

Bruker SMART APEX CCD diffractometer	3004 independent reflections
Radiation source: fine-focus sealed tube, Siemens KFFCU2K-90	2984 reflections with *I* > 2σ(*I*)
Graphite monochromator	*R*_int_ = 0.032
Detector resolution: 8.33 pixels mm^-1^	θ_max_ = 69.5°, θ_min_ = 4.2°
φ and ω scans	*h* = −8→8
Absorption correction: multi-scan (*SADABS*; Krause *et al.*, 2015)	*k* = −14→14
*T*_min_ = 0.701, *T*_max_ = 0.929	*l* = −24→22
11963 measured reflections	

### Refinement

**Table:**

Refinement on *F*^2^	Secondary atom site location: difference Fourier map
Least-squares matrix: full	Hydrogen site location: inferred from neighbouring sites
*R*[*F*^2^ > 2σ(*F*^2^)] = 0.028	H-atom parameters constrained
*wR*(*F*^2^) = 0.070	*w* = 1/[σ^2^(*F*_o_^2^) + (0.0405*P*)^2^ + 0.5066*P*] where *P* = (*F*_o_^2^ + 2*F*_c_^2^)/3
*S* = 1.11	(Δ/σ)_max_ = 0.001
3004 reflections	Δρ_max_ = 0.29 e Å^−^^3^
237 parameters	Δρ_min_ = −0.36 e Å^−^^3^
0 restraints	Absolute structure: Flack *x* determined using 1183 quotients [(*I*^+^)-(*I*^-^)]/[(*I*^+^)+(*I*^-^)] (Parsons *et al.*, 2013)
Primary atom site location: structure-invariant direct methods	Absolute structure parameter: 0.052 (4)

### Special details

**Table:**

Geometry. All e.s.d.'s (except the e.s.d. in the dihedral angle between two l.s. planes) are estimated using the full covariance matrix. The cell e.s.d.'s are taken into account individually in the estimation of e.s.d.'s in distances, angles and torsion angles; correlations between e.s.d.'s in cell parameters are only used when they are defined by crystal symmetry. An approximate (isotropic) treatment of cell e.s.d.'s is used for estimating e.s.d.'s involving l.s. planes.

### Fractional atomic coordinates and isotropic or equivalent isotropic displacement parameters (Å^2^)

**Table:**

	*x*	*y*	*z*	*U*_iso_*/*U*_eq_	
S1	0.45871 (9)	0.55630 (5)	0.77781 (3)	0.00978 (15)	
S2	0.67201 (9)	0.69687 (5)	0.85264 (3)	0.01185 (16)	
O1	0.3825 (3)	0.25928 (14)	0.82183 (9)	0.0148 (4)	
O2	0.6694 (3)	0.34297 (15)	0.80444 (9)	0.0176 (4)	
O3	0.8276 (3)	0.63677 (15)	0.82056 (9)	0.0175 (4)	
O4	0.6751 (3)	0.81443 (15)	0.84687 (9)	0.0185 (4)	
N1	0.3831 (3)	0.44084 (18)	0.81606 (11)	0.0127 (5)	
N2	0.4579 (3)	0.65675 (17)	0.82812 (11)	0.0132 (5)	
C1	0.4975 (4)	0.3476 (2)	0.81239 (12)	0.0129 (5)	
C2	0.1767 (4)	0.2920 (2)	0.82310 (13)	0.0136 (5)	
H2	0.1106	0.2809	0.7795	0.016*	
C3	0.0725 (4)	0.2347 (2)	0.87983 (13)	0.0148 (6)	
H3A	−0.0688	0.2299	0.8713	0.018*	
H3B	0.1246	0.1601	0.8867	0.018*	
C4	0.1142 (4)	0.3068 (2)	0.93859 (13)	0.0124 (5)	
C5	0.0916 (4)	0.2842 (2)	1.00541 (13)	0.0140 (6)	
H5	0.0423	0.2157	1.0198	0.017*	
C6	0.1431 (4)	0.3648 (2)	1.05088 (13)	0.0161 (6)	
H6	0.1258	0.3512	1.0968	0.019*	
C7	0.2188 (4)	0.4644 (2)	1.03076 (14)	0.0157 (6)	
H7	0.2569	0.5170	1.0628	0.019*	
C8	0.2393 (4)	0.4875 (2)	0.96324 (14)	0.0141 (5)	
H8	0.2899	0.5558	0.9488	0.017*	
C9	0.1843 (4)	0.4088 (2)	0.91814 (13)	0.0117 (5)	
C10	0.1900 (4)	0.41298 (19)	0.84277 (13)	0.0116 (5)	
H10	0.0833	0.4584	0.8234	0.014*	
C11	0.2421 (4)	0.5860 (2)	0.73290 (13)	0.0149 (6)	
H11A	0.1358	0.5997	0.7641	0.022*	
H11B	0.2089	0.5237	0.7046	0.022*	
H11C	0.2627	0.6510	0.7054	0.022*	
C12	0.6749 (4)	0.6636 (2)	0.93784 (12)	0.0112 (5)	
C13	0.6177 (4)	0.7407 (2)	0.98430 (13)	0.0136 (5)	
H13	0.5750	0.8110	0.9706	0.016*	
C14	0.6237 (4)	0.7138 (2)	1.05134 (13)	0.0153 (6)	
H14	0.5858	0.7664	1.0834	0.018*	
C15	0.6846 (4)	0.6107 (2)	1.07178 (13)	0.0157 (5)	
C16	0.7366 (4)	0.5341 (2)	1.02403 (14)	0.0152 (6)	
H16	0.7745	0.4628	1.0376	0.018*	
C17	0.7344 (4)	0.5596 (2)	0.95710 (14)	0.0147 (5)	
H17	0.7727	0.5071	0.9250	0.018*	
C18	0.6940 (5)	0.5854 (2)	1.14496 (14)	0.0231 (6)	
H18A	0.7504	0.5126	1.1514	0.035*	
H18B	0.5628	0.5871	1.1637	0.035*	
H18C	0.7752	0.6400	1.1672	0.035*	

### Atomic displacement parameters (Å^2^)

**Table:**

	*U*^11^	*U*^22^	*U*^33^	*U*^12^	*U*^13^	*U*^23^
S1	0.0116 (3)	0.0111 (3)	0.0066 (3)	−0.0007 (2)	0.0007 (2)	0.0007 (2)
S2	0.0124 (3)	0.0139 (3)	0.0093 (3)	−0.0022 (2)	0.0002 (2)	−0.0003 (2)
O1	0.0189 (10)	0.0105 (8)	0.0151 (9)	0.0019 (7)	0.0014 (7)	−0.0003 (7)
O2	0.0161 (9)	0.0186 (9)	0.0179 (9)	0.0046 (8)	0.0011 (8)	0.0004 (8)
O3	0.0116 (9)	0.0269 (10)	0.0140 (9)	−0.0032 (8)	0.0015 (8)	−0.0017 (8)
O4	0.0251 (10)	0.0161 (9)	0.0143 (9)	−0.0067 (8)	−0.0028 (8)	0.0036 (7)
N1	0.0141 (11)	0.0108 (10)	0.0133 (10)	0.0016 (9)	0.0039 (9)	0.0032 (9)
N2	0.0135 (11)	0.0141 (10)	0.0118 (10)	0.0004 (9)	0.0001 (9)	−0.0013 (9)
C1	0.0170 (14)	0.0137 (12)	0.0080 (12)	0.0023 (10)	0.0010 (10)	−0.0015 (10)
C2	0.0157 (13)	0.0129 (12)	0.0121 (12)	−0.0016 (11)	−0.0025 (11)	0.0014 (10)
C3	0.0173 (14)	0.0137 (12)	0.0134 (13)	−0.0036 (10)	−0.0014 (11)	0.0008 (11)
C4	0.0116 (11)	0.0133 (12)	0.0121 (12)	0.0020 (10)	−0.0014 (10)	0.0006 (11)
C5	0.0109 (12)	0.0164 (13)	0.0149 (13)	0.0002 (10)	0.0010 (10)	0.0059 (10)
C6	0.0122 (13)	0.0256 (14)	0.0106 (12)	0.0041 (11)	0.0015 (10)	0.0017 (11)
C7	0.0107 (13)	0.0218 (13)	0.0145 (13)	0.0024 (10)	−0.0001 (10)	−0.0054 (11)
C8	0.0114 (12)	0.0135 (12)	0.0174 (13)	0.0023 (10)	0.0033 (10)	−0.0010 (11)
C9	0.0090 (11)	0.0148 (12)	0.0111 (12)	0.0030 (10)	0.0025 (11)	0.0023 (10)
C10	0.0112 (12)	0.0120 (12)	0.0117 (12)	0.0003 (9)	0.0010 (11)	0.0030 (9)
C11	0.0168 (13)	0.0158 (12)	0.0122 (13)	−0.0026 (10)	−0.0057 (10)	0.0050 (10)
C12	0.0087 (11)	0.0168 (12)	0.0080 (11)	−0.0023 (10)	−0.0023 (10)	0.0024 (9)
C13	0.0106 (12)	0.0158 (12)	0.0142 (13)	−0.0013 (10)	−0.0004 (10)	0.0011 (11)
C14	0.0116 (12)	0.0215 (13)	0.0127 (12)	−0.0028 (10)	0.0013 (10)	−0.0013 (10)
C15	0.0111 (12)	0.0223 (13)	0.0136 (13)	−0.0054 (11)	−0.0024 (11)	0.0031 (11)
C16	0.0107 (12)	0.0156 (12)	0.0192 (13)	−0.0008 (10)	−0.0024 (10)	0.0050 (11)
C17	0.0103 (12)	0.0158 (12)	0.0180 (13)	−0.0003 (10)	0.0015 (10)	−0.0028 (11)
C18	0.0263 (16)	0.0287 (15)	0.0144 (13)	−0.0091 (12)	−0.0016 (13)	0.0049 (11)

### Geometric parameters (Å, º)

**Table:**

S1—N2	1.594 (2)	C7—C8	1.399 (4)
S1—N1	1.692 (2)	C7—H7	0.9500
S1—C11	1.782 (3)	C8—C9	1.378 (4)
S2—O4	1.4430 (19)	C8—H8	0.9500
S2—O3	1.451 (2)	C9—C10	1.523 (3)
S2—N2	1.631 (2)	C10—H10	1.0000
S2—C12	1.768 (2)	C11—H11A	0.9800
O1—C1	1.353 (3)	C11—H11B	0.9800
O1—C2	1.472 (3)	C11—H11C	0.9800
O2—C1	1.195 (3)	C12—C13	1.387 (4)
N1—C1	1.388 (3)	C12—C17	1.392 (4)
N1—C10	1.475 (3)	C13—C14	1.394 (4)
C2—C3	1.522 (4)	C13—H13	0.9500
C2—C10	1.535 (3)	C14—C15	1.391 (4)
C2—H2	1.0000	C14—H14	0.9500
C3—C4	1.506 (4)	C15—C16	1.392 (4)
C3—H3A	0.9900	C15—C18	1.511 (4)
C3—H3B	0.9900	C16—C17	1.387 (4)
C4—C5	1.386 (4)	C16—H16	0.9500
C4—C9	1.400 (4)	C17—H17	0.9500
C5—C6	1.393 (4)	C18—H18A	0.9800
C5—H5	0.9500	C18—H18B	0.9800
C6—C7	1.386 (4)	C18—H18C	0.9800
C6—H6	0.9500		
			
N2—S1—N1	110.58 (11)	C9—C8—H8	120.8
N2—S1—C11	99.43 (12)	C7—C8—H8	120.8
N1—S1—C11	98.34 (11)	C8—C9—C4	121.5 (2)
O4—S2—O3	117.24 (12)	C8—C9—C10	129.0 (2)
O4—S2—N2	106.79 (12)	C4—C9—C10	109.5 (2)
O3—S2—N2	112.27 (11)	N1—C10—C9	113.4 (2)
O4—S2—C12	107.93 (11)	N1—C10—C2	100.5 (2)
O3—S2—C12	108.03 (12)	C9—C10—C2	103.0 (2)
N2—S2—C12	103.67 (12)	N1—C10—H10	113.0
C1—O1—C2	110.39 (19)	C9—C10—H10	113.0
C1—N1—C10	110.0 (2)	C2—C10—H10	113.0
C1—N1—S1	119.14 (18)	S1—C11—H11A	109.5
C10—N1—S1	129.59 (17)	S1—C11—H11B	109.5
S1—N2—S2	114.97 (13)	H11A—C11—H11B	109.5
O2—C1—O1	124.1 (2)	S1—C11—H11C	109.5
O2—C1—N1	127.5 (2)	H11A—C11—H11C	109.5
O1—C1—N1	108.5 (2)	H11B—C11—H11C	109.5
O1—C2—C3	110.0 (2)	C13—C12—C17	121.0 (2)
O1—C2—C10	102.1 (2)	C13—C12—S2	119.9 (2)
C3—C2—C10	106.1 (2)	C17—C12—S2	119.0 (2)
O1—C2—H2	112.7	C12—C13—C14	119.2 (2)
C3—C2—H2	112.7	C12—C13—H13	120.4
C10—C2—H2	112.7	C14—C13—H13	120.4
C4—C3—C2	103.5 (2)	C15—C14—C13	120.7 (3)
C4—C3—H3A	111.1	C15—C14—H14	119.6
C2—C3—H3A	111.1	C13—C14—H14	119.6
C4—C3—H3B	111.1	C14—C15—C16	118.9 (2)
C2—C3—H3B	111.1	C14—C15—C18	119.3 (3)
H3A—C3—H3B	109.0	C16—C15—C18	121.9 (3)
C5—C4—C9	120.2 (2)	C17—C16—C15	121.3 (2)
C5—C4—C3	128.9 (2)	C17—C16—H16	119.3
C9—C4—C3	110.8 (2)	C15—C16—H16	119.3
C4—C5—C6	118.1 (2)	C16—C17—C12	118.8 (2)
C4—C5—H5	120.9	C16—C17—H17	120.6
C6—C5—H5	120.9	C12—C17—H17	120.6
C7—C6—C5	121.6 (2)	C15—C18—H18A	109.5
C7—C6—H6	119.2	C15—C18—H18B	109.5
C5—C6—H6	119.2	H18A—C18—H18B	109.5
C6—C7—C8	120.1 (3)	C15—C18—H18C	109.5
C6—C7—H7	120.0	H18A—C18—H18C	109.5
C8—C7—H7	120.0	H18B—C18—H18C	109.5
C9—C8—C7	118.3 (2)		
