# Supplementary figures and images for: Crystal structure of 4-methyl-N-{(E)-meth­yl[(3aR,8aS)-2-oxo-3,3a,8,8a-tetra­hydro-2H-indeno­[1,2-d][1,3]oxazol-3-yl]-λ4-sulfanyl­idene}benzene­sulfonamide
Source: Acta Crystallogr E Crystallogr Commun. 2015 Dec 31;71(Pt 12):o1097–8. doi: 10.1107/S2056989015024779 (PMC4719998; doi:10.1107/S2056989015024779)

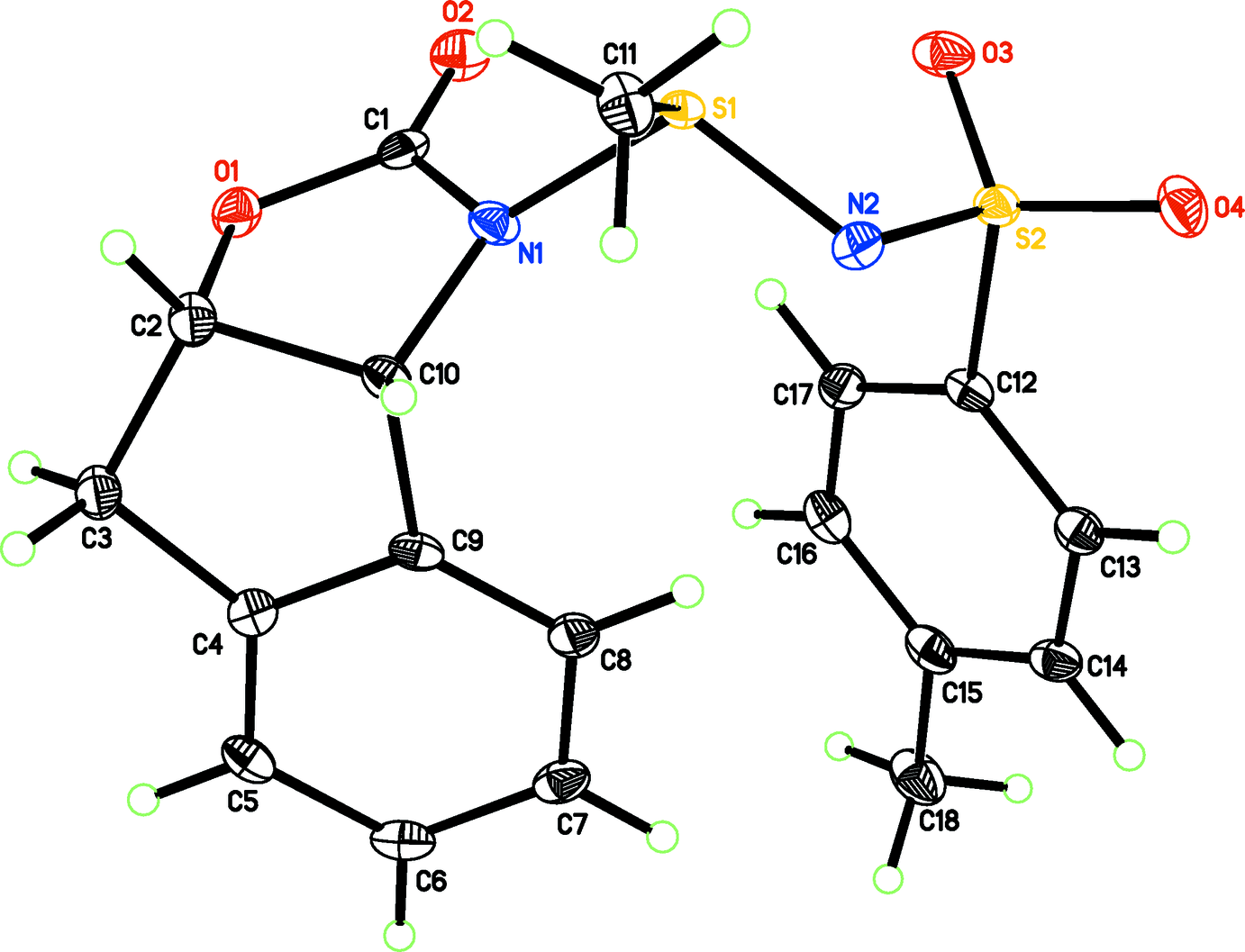

Supplement: Supplementary file 4 [file e-71-o1097-fig1.tif]
